# Notopterol Ameliorates Hyperuricemia-Induced Cardiac Dysfunction in Mice

**DOI:** 10.3390/ph16030361

**Published:** 2023-02-27

**Authors:** Qian Wang, Dewei Peng, Bingyu Huang, Lintong Men, Tao Jiang, Shengqi Huo, Moran Wang, Junyi Guo, Jiagao Lv, Li Lin

**Affiliations:** 1 Division of Cardiology, Department of Internal Medicine, Tongji Hospital, Tongji Medical College, Huazhong University of Science and Technology, Wuhan 430074, China; 2 Department of Geriatrics, Tongji Hospital, Tongji Medical College, Huazhong University of Science and Technology, Wuhan 430074, China

**Keywords:** notopterol, hyperuricemia, inflammation, pyroptosis, P2X7R, NLRP3

## Abstract

Notopterol is a naturally occurring furanocoumarin compound found in the root of Notopterygium incisum. Hyperuricemia involves the activation of chronic inflammation and leads to cardiac damage. Whether notopterol has cardioprotective potential in hyperuricemia mice remains elusive. The hyperuricemic mouse model was constructed by administration of potassium oxonate and adenine every other day for six weeks. Notopterol (20 mg/kg) and allopurinol (10 mg/kg) were given daily as treatment, respectively. The results showed that hyperuricemia dampened heart function and reduced exercise capacity. Notopterol treatment improved exercise capacity and alleviated cardiac dysfunction in hyperuricemic mice. P2X7R and pyroptosis signals were activated both in hyperuricemic mice and in uric acid-stimulated H9c2 cells. Additionally, it was verified that inhibition of P2X7R alleviated pyroptosis and inflammatory signals in uric acid-treated H9c2 cells. Notopterol administration significantly suppressed expression levels of pyroptosis associated proteins and P2X7R in vivo and in vitro. P2X7R overexpression abolished the inhibition effect of notopterol on pyroptosis. Collectively, our findings suggested that P2X7R played a critical role in uric acid-induced NLRP3 inflammatory signals. Notopterol inhibited pyroptosis via inhibiting the P2X7R/NLRP3 signaling pathway under uric acid stimulation. Notopterol might represent a potential therapeutic strategy against pyroptosis and improve cardiac function in hyperuricemic mice.

## 1. Introduction

Excessive accumulation of serum uric acid leads to hyperuricemia, which is considered an independent risk factor for cardiovascular diseases [1]. Hyperuricemia mainly occur in higher primates, including in humans, because the gene encoding urate oxidase is inactivated in higher primates [2]. Elevated serum uric acid has been found to be associated with adverse outcomes in heart failure patients [3]. The most common lifestyle risk factor for hyperuricosuria is dietary imbalance, especially consumption of high amounts of fructose, purine meats and beer [4]. The incidence of hyperuricemia was substantial and with rising trends particularly among men [5]. Thus, it is urgent to investigate the mechanism and applicable treatment for cardiac damage induced by hyperuricemia.

Natural plants and compounds are widely used to treat various cardiovascular disorders, due in part to their low toxicity and high effectiveness [6]. Notopterygium incisum (Qiang Huo) has been an extensively used traditional Chinese medicine for a long time. It has been used to treat rheumatism, arthralgia headaches and other symptoms. In addition, it has anti-inflammatory and cardioprotective effects [7]. Notopterol is a naturally occurring furanocoumarin compound which is present in the root of Notopterygium incisum. Moreover, notopterol is one of the active components of Notopterygium incisum [8,9]. The chemical structure of notopterol is shown in Supplementary Figure S1. Notopterol has anti-inflammatory and anti-proliferative properties in the pulmonary arteries and attenuates pulmonary hypertension in rats [10]. Additionally, notopterol relieves inflammation by inhibiting the JAK-STAT signaling pathway in rheumatoid arthritis mice models [11]. Notopterol administration suppresses the expression of inflammatory cytokines, such as interleukin-6 (IL-6), tumor necrosis factor-α (TNF-α), and interleukin-1β (IL-1β) [10,11]. However, it has not yet been clarified whether notopterol exerts the potential to ameliorate cardiac dysfunction induced by hyperuricemia. In addition, the targets of notopterol have not been clearly elucidated.

Hyperuricemia contributes to chronic inflammation activation. Pyroptosis is a highly programmed inflammatory cell death that is dependent on caspase-1 activation in inflammasomes [12,13]. Pyroptosis has been shown to play an essential role in the pathogenesis of cardiac damage induced by soluble uric acid [14,15]. The core of pyroptosis is to activate the NLRP3 inflammasome, which triggers the activation of caspase-1 and the maturation and secretion of proinflammatory cytokines such as IL-1β and interleukin-18 (IL-18) [16]. The purinergic receptor P2X7 (P2X7R) is a purinergic signaling receptor and its function in NLRP3 inflammasome activation has been highlighted [17]. Increased P2X7R expression has been shown to play pathological roles in various cardiovascular diseases [18,19]. However, the role of P2X7R in cardiac damage induced by hyperuricemia has yet to be elucidated. Allopurinol is a commonly clinically used urate-lowering drug. Allopurinol treatment was used to explore whether uric acid-lowering treatment alone could alleviate heart dysfunction in hyperuricemic mice. In this study, we assessed the effect of P2X7R in promoting pyroptosis under uric acid stimulation, and investigated whether notopterol could ameliorate cardiac dysfunction induced by hyperuricemia, along with its possible mechanisms.

## 2. Results

### 2.1. Notopterol Increased Exercise Capacity and Attenuated Cardiac Dysfunction in Hyperuricemic Mice

**Figure 1:**
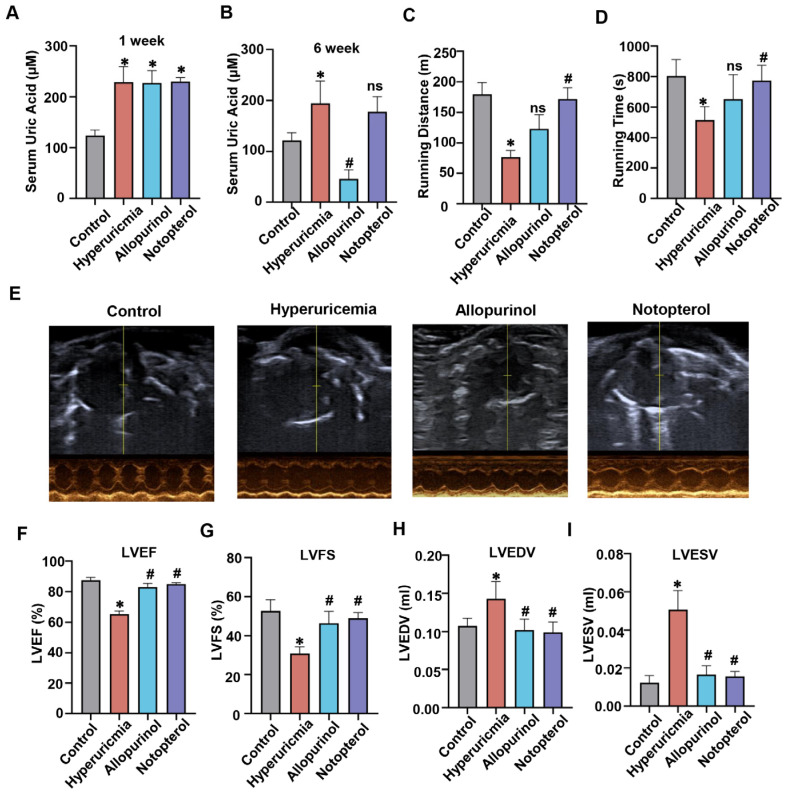
Notopterol improved exercise capacity and alleviated hyperuricemia-induced cardiac dysfunction. (**A**) Serum uric acid was measured at the first week after hyperuricemia induction (n = 5 mice/group). (**B**). Serum uric acid was measured at the sixth week after hyperuricemia induction (n = 5 mice/group) (**C**) Running distance of treadmill fatigue test performed at the sixth week after hyperuricemia induction (n = 5 mice/group). (**D**) Running time of treadmill fatigue test performed at the sixth week after hyperuricemia induction (n = 5 mice/group). (**E**) The representative echocardiographic images from the parasternal short axis view which were used to analyzed the cardiac function of the mice at the sixth week after hyperuricemia induction. The vertical line is the reference line for the position of the cardiac ultrasound probe. (**F**) Left ventricular ejection fraction (LVEF), (**G**) Left ventricular fractional shortening (LVFS), (**H**) Left ventricular volumes at end diastole (LVEDV) (**I**) and systole (LVESV) measured at the sixth week after hyperuricemia induction (n = 5 mice/group). Data represent means ± S.E.M. The symbol “∗” represents *p* < 0.05 vs. control. # *p* < 0.05 vs. hyperuricemia group. The “ns” stands for “no significance”.

To investigate the impact of allopurinol and notopterol in vivo, we treated mice with allopurinol (10 mg/kg) or notopterol (20 mg/kg) from the second week post hyperuricemia induction for four weeks. The flowchart showing the experimental process for modeling mice can be seen in Supplementary Figure S2A. One week after the induction of hyperuricemia, the serum uric acid level was significantly increased compared with control group, indicating that a hyperuricemic mouse model had been successfully established (Figure 1A). At the end of the experiment, the hyperuricemic mice showed higher serum uric acid in comparison to that of control (Figure 1B). Allopurinol treatment significantly reduced serum uric acid level. Compared with hyperuricemia mice, notopterol administration did not affect serum uric acid level. Treadmill fatigue tests were performed to detect exercise capacity. Hyperuricemic mice displayed impaired exercise capacity with shorter running distance and running time compared to control group (Figure 1C,D). Allopurinol treatment increased running time and running distance, but the differences were not significant. Notopterol administration markedly improved exercise capacity as evidenced by greater distance and duration time of running relative to the hyperuricemia group.

To investigate the effect of hyperuricemia on cardiac function, we assessed the cardiac function using echocardiography at two different time points. Two weeks after the induction of hyperuricemia, left ventricular ejection fraction (LVEF) and left ventricular fractional shortening (LVFS) rate in hyperuricemic mice were lower than those in the control group (Supplementary Figure S2B,C). Left ventricular volumes at end diastole (LVEDV) and left ventricular volumes at end systole (LVESV) showed no significant difference between hyperuricemic mice and control mice (Supplementary Figure S2D,E). Using echocardiography, we also assessed the protective effect of notopterol and lowing uric acid on the cardiac function of hyperuricemic mice. Figure 1E showed the representative echocardiographic images from the parasternal short axis view. Hyperuricemia remarkably decreased LVEF and LVFS rate, but increased LVEDV and LVESV at the sixth week (Figure 1F–I). Comparing with hyperuricemic mice, allopurinol and notopterol treatment for four weeks both significantly improved LVEF and LVFS, and reduced LVEDV and LVESV levels. Taken together, these results indicated that notopterol has the potential to alleviate hyperuricemia-induced cardiac dysfunction in mice.

### 2.2. Notopterol Suppressed Inflammatory Cytokine Production in the Cardiac Tissue of Hyperuricemic Mice

**Figure 2:**
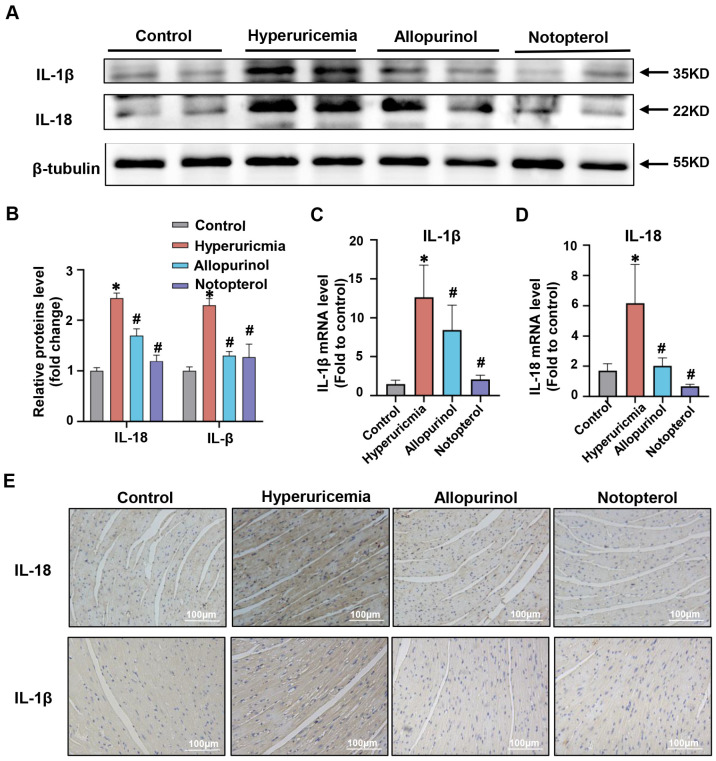
Notopterol attenuated inflammatory cytokine production in cardiac of hyperuricemia mice. (**A**) The expression level of IL-18 and IL-1β in heart tissues were analyzed by Western blot. (**B**) The quantitative statistical diagrams of IL-1β and IL-18 detected by Western blot (n = 5 mice/group). (**C**) The mRNA expression levels of IL-18 (**D**)and IL-1β in heart tissues were analyzed by RT-PCR. (**E**) Representative immunohistochemical images of IL-18 (brown) and IL-1β (brown) in mice heart tissues (n = 5 mice/group). Scale bar: 100 µm. Data represent means ± S.E.M. The symbol “∗” represents *p* < 0.05 vs. control. # *p* < 0.05 vs. hyperuricemia group.

Excessive inflammatory cytokine production plays a significant role in the development of cardiac injury. IL-1β and IL-18 are vital proinflammatory cytokines in the NLRP3 inflammasome-dependent pyroptosis signaling pathway. Previous studies have shown that IL-1β could be induced in the cardiac of hyperuricemia rats and promoted cardiac damage [15]. Consistent with previous findings, the higher level of IL-1β was detected in the cardiac tissue of hyperuricemic mice compared with the control group (*p* < 0.05) (Figure 2A,B). Moreover, IL-18 level in the cardiac of hyperuricemia mice was also elevated (Figure 2A,B). Notopterol and allopurinol treatment significantly reduced protein levels of IL-1β and IL-18 in cardiac tissue (Figure 2A,B). We determined the mRNA expression levels of IL-1β and IL-18 by quantitative real-time PCR. There was an ameliorative effect of allopurinol and notopterol on mRNA levels of IL-1β and IL-18 in cardiac tissue of hyperuricemic mice (Figure 2C,D). Immunohistochemical staining was also used to clarify the effects of allopurinol and notopterol on IL-1β and IL-18 in heart tissue. These results validated that allopurinol at 10 mg/kg and notopterol at 20 mg/kg both attenuated inflammation cytokine production in the cardiac tissue of hyperuricemic mice.

### 2.3. Notopterol Ameliorated Cardiac Pyroptosis in Hyperuricemic Mice

**Figure 3:**
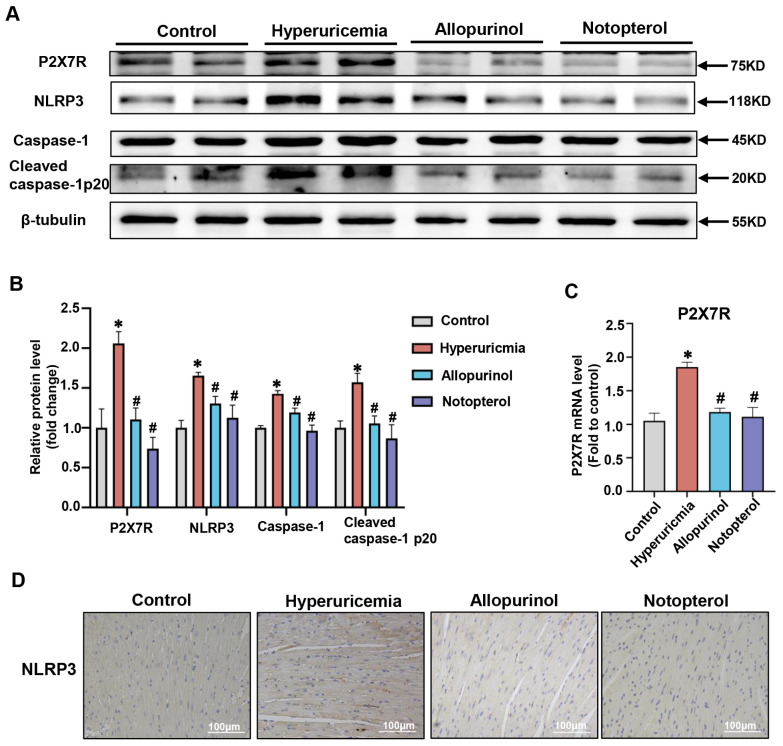
NLRP3 and P2X7R were upregulated in heart tissue of hyperuricemic mice, notopterol suppressed P2X7R and NLRP3 inflammasome signals. (**A**) The expression level of P2X7R, NLRP3, Caspase-1 and Cleaved caspase-1 p20 in heart tissues were analyzed by Western blot. (**B**) The quantitative statistical diagrams of P2X7R, NLRP3, Caspase1, and Cleaved caspase-1 p20 detected by Western blot (n = 5 mice/group). (C) The mRNA level of P2X7R in heart tissues were analyzed by RT-PCR. (**D**) Representative immunohistochemical images of NLRP3 (brown) in mice heart tissues. Scale bar: 100 µm. Data represent means ± S.E.M. The symbol “∗” represents *p* < 0.05 vs. control. # *p* < 0.05 vs. hyperuricemia group.

Pyroptosis has been shown to play a vital role in the pathogenesis of cardiac damage induced by hyperuricemia [15]. To determine whether notopterol could attenuate hyperuricemia-induced pyroptosis, we examined pyroptosis-associated protein expression levels in the cardiac tissue of hyperuricemic mice. NLRP3, caspase-1 and cleaved caspase-1 p20 were dramatically increased in hyperuricemic mice while allopurinol (10 mg/kg) and notopterol (20 mg/kg) suppressed their expressions (Figure 3A,B). The mRNA expression of P2X7R increased in cardiac tissues of hyperuricemic mice. Allopurinol and notopterol administration decreased the expression of P2X7R mRNA level in cardiac tissue. These results suggest that notopterol could affect the transcription level of P2X7R in heart tissue (Figure 3C). Immunohistochemistry revealed that allopurinol and notopterol suppressed the expression of NLRP3 in heart tissue of hyperuricemic mice (Figure 3D). The P2X7R is a regulator of pyroptosis [19]. P2X7R expression was increased in heart tissue of hyperuricemic mice and inhibited by allopurinol and notopterol administration (Figure 3A,B). These results indicate that pyroptosis and P2X7R were both upregulated in hyperuricemic mice and that notopterol suppressed pyroptosis and P2X7R signals.

### 2.4. Notopterol Attenuated Inflammatory Cytokine Production and Pyroptosis Induced by Uric Acid in H9C2 Cells

**Figure 4:**
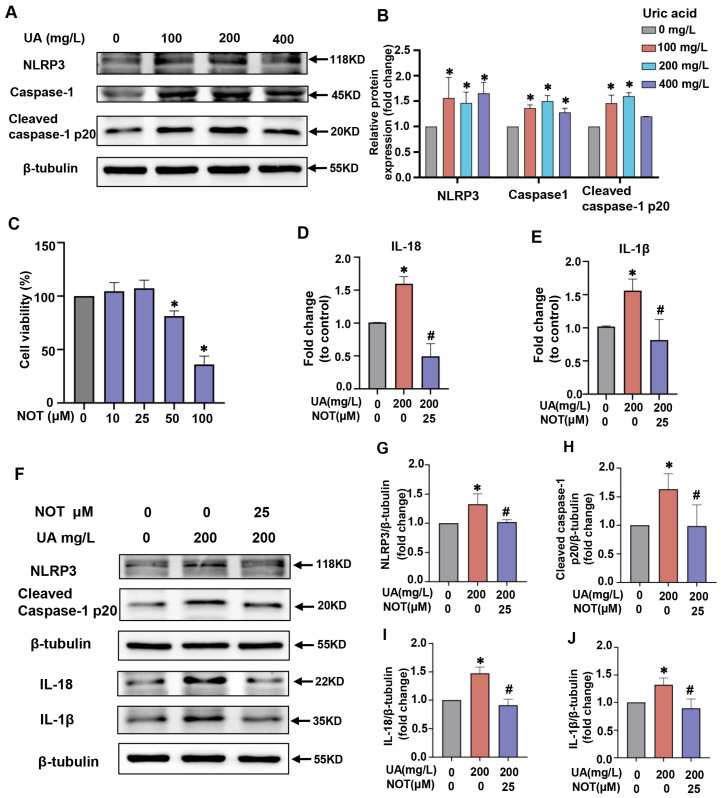
Notopterol attenuated inflammatory cytokine and pyroptosis induced by uric acid in H9c2 cells. (**A**) After H9c2 cells were stimulated with uric acid (0, 100, 200 and 400 mg/L) for 24 h, the protein levels of NLRP3, Caspase-1 and Cleaved caspase-1 p20 were determined by western blot analysis. (**B**) The quantitative statistical diagrams of NLRP3, Caspase-1 and Cleaved caspase-1 p20 detected by Western blot (n = 3/group). (**C**) Cell viability of H9c2 cells were measured using cck8 assay after treatment of various concentrations notopterol (0, 10, 25, 50 and 100 μM) for 24 h. (**D**,**E**) H9c2 cells were treated with uric acid (200 mg/L) with/without notopterol (25 μM) administration for 24 h. The mRNA expression levels of IL-18 and IL-1β were analyzed by RT-PCR (n = 3/group). (**F**) and the protein expression levels of NLRP3, Cleaved caspase-1 p20, IL-18 and IL-1β were analyzed by Western blot. (**G**–**J**) The quantitative statistical diagrams of NLRP3, Caspase-1 and Cleaved caspase-1 p20 detected by Western blot (n = 3/group). Data represent means ± S.E.M. ∗ *p* < 0.05 vs. control; # *p* < 0.05 vs. uric acid treatment. UA, Uric acid. NOT, notopterol.

To investigate the effects of uric acid on pyroptosis in vitro, H9c2 cells were exposed to uric acid at different concentrations for 24 h. Compared with control group, uric acid stimulation increased NLRP3, caspase1, and cleaved caspase-1 p20 expression in H9c2 cells. At the concentration of 200 mg/L, the maximum protein expression level of caspase1, and cleaved caspase-1 p20 was observed and a concentration of 200 mg/L for 24 h was chosen for further study (Figure 4A,B).To select the appropriate concentration of notopterol for further experiments, we detected the cytotoxicity of notopterol by the CCK8 assay. As shown in Figure 4C, 25 μM notopterol treatment or a lower concentration had no obvious effect on cytotoxicity. Therefore, 25 μM was chosen for further experiments in H9c2 cells. RT-PCR results showed that notopterol inhibited the uric acid-triggered proinflammatory cytokine production including IL-1β and IL-18 (Figure 4D,E). In parallel, we found that uric acid upregulated the protein expression levels of NLRP3, cleaved caspase-1, IL-1β and IL-18, which were inhibited by notopterol (Figure 4F–J).

### 2.5. P2X7R Was Upregulated in Uric Acid-Stimulated H9C2 Cells and Notopterol Suppressed P2X7R Signaling

**Figure 5:**
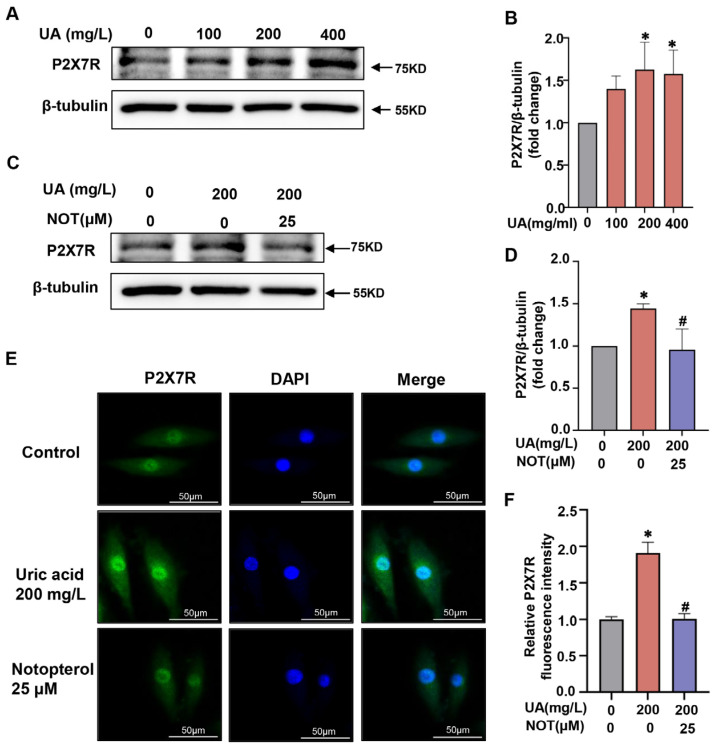
P2X7R was upregulated in uric acid induced H9C2 cells, notopterol suppressed P2X7R signals. (**A**) The expression level of P2X7R was determined by western blot analysis after treatment of various concentrations of uric acid (0, 100, 200, and 400 mg/L) for 24 h in H9c2 cells. (**B**) The quantitative statistical diagrams of P2X7R detected by Western blot (n = 3/group). (**C**) H9c2 cells were treated with uric acid (200 mg/L) with/without notopterol (25 μM) administration for 24 h. The expression level of P2X7R was determined by western blot. (**D**) The quantitative statistical diagrams of P2X7R detected by Western blot (n = 3/group). (**E**) After H9c2 cells were treated with uric acid (200 mg/L) with/without notopterol (25 μM) administration for 24 h, P2X7R was detected by fluorescence analysis in H9c2 cells. Green: P2X7R. Blue, DAPI. (**F**) Fluorescence intensity quantification by image J software (n = 3/group). Scale bar = 50 μm. Data represent means ± S.E.M. ∗ *p* < 0.05 vs. control; # *p* < 0.05 vs. uric acid treatment. UA, Uric acid. NOT, notopterol.

We further explored the mechanism by which notopterol alleviated pyroptosis in uric acid-stimulated H9c2 cells. Pyroptosis can be activated through multiple pathways including P2X7R signaling. However, whether P2X7R was involved in the pyroptosis and inflammation of H9c2 cells induced by uric acid remains unclear. Uric acid treatment increased P2X7R expression levels in a concentration-dependent manner in H9c2 cells (Figure 5A,B). Consistently, the P2X7R was significantly upregulated under uric acid stimulation and was restored by notopterol (25 μM) treatment (*p* < 0.05) (Figure 5C,D). Immunofluorescence was also utilized to verify the effect of uric acid and notopterol on P2X7R. In agreement with the western blot experiment, the P2X7R expression level induced by uric acid was suppressed by notopterol (*p* < 0.05) (Figure 5E,F).

### 2.6. P2X7R Regulated NLRP3 Inflammasome Signals in H9C2 Cells

**Figure 6:**
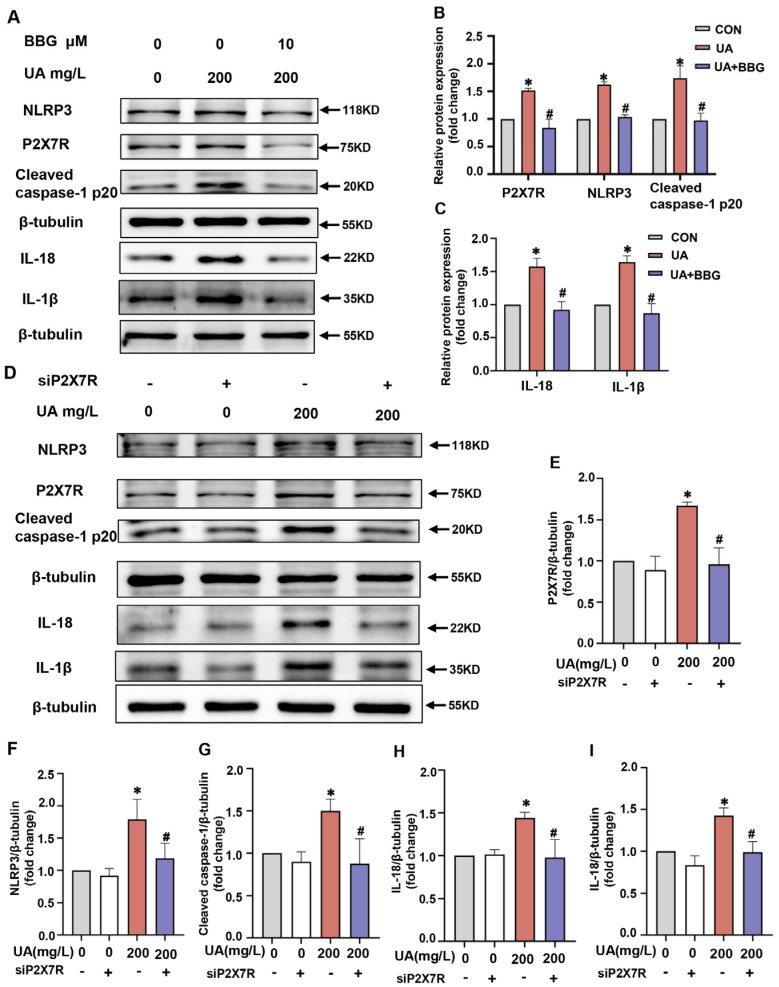
P2X7R regulated NLRP3 inflammasome signals and inflammatory cytokines in H9C2 cells. (**A**) H9c2 cells were treated with uric acid (200 mg/L) with/without BBG (10 μM) administration for 24 h. The expression level of P2X7R, NLRP3, Cleaved caspase-1 p20, IL-18 and IL-1β were determined by western blot. (**B**,**C**) The quantitative statistical diagrams of P2X7R, NLRP3, Caspase-1 and Cleaved caspase-1 p20 detected by Western blot (n = 3/group). (**D**) H9c2 cells were transfected with control siRNA and siP2X7R, and then treated with/without uric acid (200 mg/L) for 24 h. The expression level of P2X7R, NLRP3, Cleaved caspase-1 p20, IL-18 and IL-1β were determined by western blot. (**E**–**I**) The quantitative statistical diagrams of P2X7R, NLRP3, Caspase-1 and Cleaved caspase-1 p20 detected by Western blot (n = 3/group). Data represent means ± S.E.M. ∗ *p* < 0.05 vs. control; # *p* < 0.05 vs. uric acid treatment. UA, Uric acid. NOT, notopterol.

To further verify that P2X7R plays a role in uric acid-induced pyroptosis in H9c2 cells, we explored whether blocking P2X7R could prevent pyroptosis. First, we evaluated the effects of the selective P2X7R inhibitor, Brilliant Blue G (BBG). BBG (10 μM) significantly suppressed the protein expression of P2X7R in uric acid-stimulated H9c2 cells. Moreover, BBG markedly inhibited pyroptosis related protein levels including NLRP3, cleaved caspase-1 p20, IL-18 and IL-1β under uric acid stimulation in H9c2 cells (Figure 6A–C). Next, we applied P2X7R siRNA (siP2X7R) to knock down P2X7R and then examined the pyroptosis-related protein levels after uric acid treatment. SiP2X7R transfection decreased P2X7R expression level compared with the control siRNA group, but the difference was not significant (Figure 6D,E). Our results demonstrated that siP2X7R markedly inhibited the levels of P2X7R induced by uric acid. Notably, the protein expression levels of NLRP3, cleaved caspase-1 p20, IL-18 and IL-1β induced by uric acid were markedlyinhibited by siP2X7R (Figure 6D,F–I). These results indicated that P2X7R might play an essential role in uric acid-induced pyroptosis in H9c2 cells.

### 2.7. Notopterol Alleviated Uric Acid-Induced Pyroptosis via Regulating P2X7R/NLRP3 Signaling

**Figure 7:**
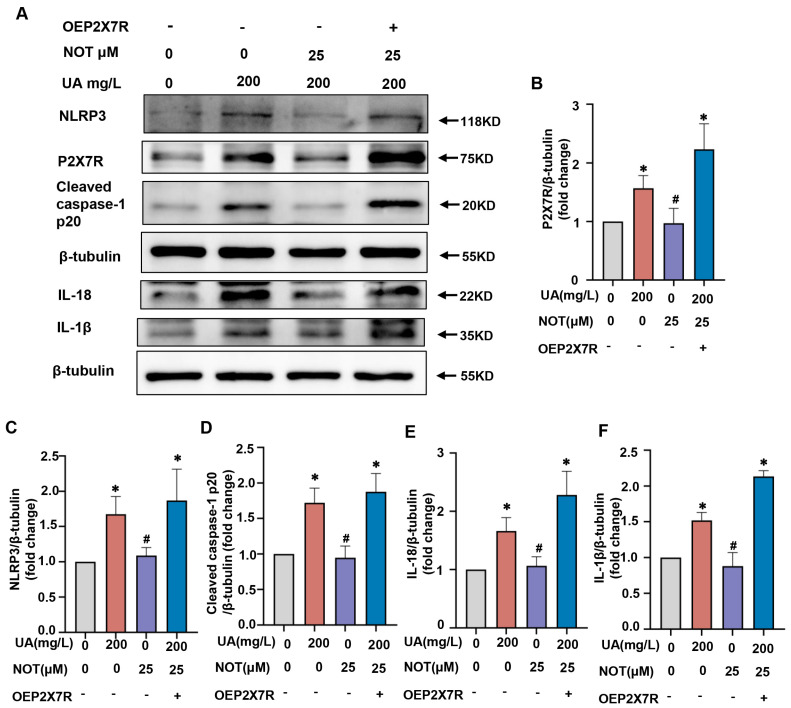
Notopterol alleviated uric acid induced pyroptosis partially via regulating P2X7R/NLRP3 axis. (**A**) H9c2 cells were transfected with control plasmid or oeP2X7R, and then treated with uric acid (200 mg/L) with or without notopterol (25 μM) treatment for 24 h. The expression levels of P2X7R, NLRP3, Cleaved caspase-1 p20, IL-18 and IL-1β were determined by western blot. (**B**–**F**) The quantitative statistical diagrams of P2X7R, NLRP3, Cleaved caspase-1 p20, IL-18 and IL-1β detected by Western blot (n = 3/group). Data represent means ± S.E.M. ∗ *p* < 0.05 vs. control; # *p* < 0.05 vs. uric acid treatment. UA, Uric acid. NOT, notopterol.

Next, we investigated whether notopterol inhibited pyroptosis partly by suppressing P2X7R expression level. The P2X7R overexpression plasmid (oeP2X7R) was used to overexpress P2X7R in H9c2 cells, and pyroptosis-related protein levels were evaluated. Our results revealed that P2X7R overexpression markedly increased the levels of P2X7R (*p* < 0.05) (Figure 7A,B). NLRP3, cleaved caspase-1 p20, IL-18 and IL-1β induced by uric acid were inhibited by notopterol treatment and P2X7R overexpression reversed the expression levels of those proteins (*p* < 0.05) (Figure 7A–F). These results revealed that notopterol alleviated uric acid induced pyroptosis partially via regulating P2X7R/NLRP3 signaling.

## 3. Discussion

Hyperuricemia is a metabolic disorder characterized by elevated blood uric acid level [20]. There is growing evidence suggesting that hyperuricemia contributes to the aggravation of cardiovascular diseases. Uric acid exerts an antioxidative property under certain conditions, but mechanism under which uric acid linked to cardiac diseases have not been fully elucidated [21]. In the present study, we found that notopterol alleviated cardiac inflammatory response, cardiac pyroptosis, exercise intolerance and cardiac dysfunction induced by hyperuricemia. Moreover, we found that notopterol inhibited NLRP3 inflammasome activation and inflammatory cytokine production via suppressing P2X7R in H9c2 cells. To our knowledge, the inhibition effect of notopterol on P2X7R expression has not been previously reported. Notopterol might represent a potential therapeutic strategy against NLRP3 inflammasome activation and improve cardiac function in hyperuricemic mice.

In humans, uric acid is the final product of purine metabolism [2]. Mouse models of hyperuricemia have been widely used by administration of adenine and potassium oxonate [22,23]. The high serum uric acid level verified the successful establishment of our hyperuricemia mouse model in this study. Impaired exercise capacity is an important predictor of poor prognosis in patients with heart failure. Our results showed that hyperuricemic mice displayed impaired exercise capacity. Allopurinol treatment normalized heart function, and it also improved running time and running distance of hyperuricemic mice, though the improvement was not significant. Notopterol administration significantly ameliorated cardiac function and exercise performance. Exercise capacity examination belongs to the category of behavioral experiments, which could be affected by multiple factors including heart dysfunction, motor neuron impairment, skeletal muscle impairment, accumulation of metabolites induced-muscle fatigue and reduced oxygen transport capacity [24,25,26,27]. Currently, the mechanism of the exercise capacity reduction of hyperuricemic mice remains to be elucidated. The improvement of cardiac function by allopurinol or notopterol might partly account for the amelioration of exercise capacity of hyperuricemic mice. A previous study identified that allopurinol treatment reduced BNP concentrations but could not improve exercise intolerance of heart failure patients in clinical trials [27]. One possibility is that the slight decrease in hemoglobin caused by allopurinol and the resulting decrease in oxygen supply to exercise muscles offset the benefits of improved cardiac function [27]. However, since we did not preserve the plasma of mice, the hemoglobin concentration could not be detected in this study. Obviously, the possible different effects of allopurinol and notopterol on motor neuron, skeletal muscle, and muscle fatigue could not be excluded.

Previous studies demonstrated that high levels of circulating uric acid could induce cardiac dysfunction in mice or rat hyperuricemia models [15,27,28]. Consistent with this literatures, cardiac function was impaired in hyperuricemia mice evaluated by echocardiography. As early as two weeks after induction of hyperuricemia, hyperuricemic mice had lower LVEF and reduced LVFS compared with control group, and the LVEDV and LVESV of hyperuricimic mice were not significantly different from mice in the control group. At the sixth week of model induction, hyperuricemia remarkably decreased LVEF and LVFS, but increased LVEDV and LVESV. These results suggesting that the continuing damage to heart function would develop if hyperuricemia was not treated. Several mechanisms may be involved in the cardiac function induced by hyperuricemia. Some researchers suggest that elevated uric acid is a direct trigger of cardiomyocytes injury, subsequently promoting cardiac pathological change [15,29]. Uric acid has been shown to be able to promote NLRP3 inflammasome activation and then induce cardiomyocyte apoptosis [15]. NLRP3 inflammasome was activated in cardiac tissue of hyperuricemic mice in our study, which might participate in cardiomyocyte damage and cardiac dysfunction. Moreover, vascular endothelial damage induced by hyperuricemia could play a role in cardiovascular diseases. Inflammation induced by uric acid could promote microvascular dysfunction, which could result in deposition of collagen with reduced contractile ability of myocardium, developing into cardiac dysfunction [30]. In our study, inflammatory cytokines were elevated in the cardiac tissue of hyperuricemic mice, including IL-1β and IL-18. The serum levels of IL-6, TNF-α, and IL-8 have been reported to increased in hyperuricemic mice models [31,32]. Some researchers also consider that hyperuricemia-induced cardiac dysfunction could be related to the reactive oxygen species produced by xanthine oxidase-mediated purine metabolism [30]. Moreover, the accumulation of uric acid had direct effects on cardiomyocytes and added stress to the heart, which could contribute to heart failure under hyperuricemia conditions [21]. The most frequent complication of hyperuricemia is kidney impairment [33]. Many studies assumed that kidney impairment could activate the renin-angiotensin system, increases angiotensin II levels and lead to cardiac damage. The role of other possible mechanisms of action in cardiac damage induced by hyperuricemia requires further investigation. Furthermore, treatment with allopurinol and notopterol from the second week post-hyperuricemia induction for four weeks attenuated cardiac dysfunction and suppressed NLRP3 activation and inflammatory cytokine production in cardiac tissue of hyperuricemic mice. These resuts suggesting that the cardiac damage in hyperuricemic mice could be reversible in the early stage of hyperuricemia induction, and treatment with allopurinol and notopterol after a period of exposure to hyperuricemia could still have beneficial effects on the heart. Allopurinol is a uric acid-lowering drug and is clinically suggested as cardio protectant [34,35,36]. However, notopterol administration did not affect serum uric acid level. These results indicated that notopterol might act by other mechanisms rather than by reducing serum uric acid.

NLRP3 inflammasome activation triggers the cleavage of pro-IL-1β and pro-IL-18, which are mediated by cleaved caspase-1, and secretes inflammatory cytokines to promote the further inflammatory process. Simultaneously, cells undergo pyroptosis. In this study, we found that NLRP3, cleaved caspase-1 were increased in the heart tissue of hyperuricemia mice. NLRP3 inflammasome has been reported to promote cardiac fibrosis and cardiomyocyte injury, and impair cardiac contractile function [37]. Notopterol administration inhibited NLRP3 inflammasome activation and inflammatory cytokine production in cardiac tissue, and improved cardiac function. Moreover, our results showed that the expression of P2X7R increased in cardiac tissue of hyperuricemic mice. NLRP3 inflammasome activation has been proved to play an essential role in the development of cardiac damage induced by hyperuricemia [14,15]. To the best of our knowledge, the role of P2X7R in cardiac dysfunction induced by hyperuricemia has never been reported. To the best of our knowledge, the role of P2X7R in cardiac dysfunction induced by hyperuricemia has never been reported. Obviously, there are many other possible pathophysiological mechanisms linking hyperuricemia and cardiac damage, including oxidative stress, endothelial dysfunction and insulin resistance [30].

P2X7R is a unique purinergic receptor with proinflammatory functions and has been found to be expressed in a variety of cell types including H9c2 cells [38]. We found that the expression of P2X7R was increased both in cardiac tissue of hyperuricemic mice and similarly in H9c2 cells induced by uric acid. Notopterol treatment suppressed the P2X7R and NLRP3 inflammasome signals in vivo and in vitro. P2X7R is a potent activator of NLRP3 inflammasome signaling and an activator of inflammatory cytokine release [39,40]. Consistent with previous studies, our results verified that NLRP3 inflammasome signals could be inhibited by BBG, a specific inhibitor of P2X7R. The basal expression level of P2X7R was low in H9c2 cells. Although siP2X7R transfection partially reduced the basal level of P2X7R expression in H9c2 cells, the difference was not significant. The inhibition effect of siP2X7R and notopterol on P2X7R expression could be obviously observed under uric acid stimulation. These results indicated that siP2X7R and notopterol suppressed the increase of P2X7R protein level induced by uric acid. Mechanistically, we found that notopterol alleviated uric acid induced pyroptosis partially via regulating P2X7R/NLRP3 signals. Overexpression of P2X7R abolished the inhibition effect of notopterol on NLRP3 inflammasome activation. Currently, the mechanism that regulates expression of P2X7R is not fully understood. Previous studies have found that P2X7R expression could be regulated by intracellular Cl- levels in monocytes [41]. Increased miR150-5p subsequently inhibited P2X7R expression in oxLDL-induced macrophages [42]. P2X7R level could also be regulated by microRNA-216b in human breast cancer [43]. Moreover, NFATc1 is a transcription factor of P2X7R in SaOS2 osteoblastic-like cells [44]. Notopterol decreased the P2X7R mRNA level in heart tissue of hyperuricemic mice, which indicated that notopterol might inhibit P2X7R gene transcription to reduce P2X7R levels. However, the exact underlying mechanism remains elusive, requiring further studies. The deregulation of pyroptosis is involved in the pathogenesis of various diseases, including cancer, nervous system diseases, cardiovascular diseases, autoimmune diseases and infectious diseases [45,46,47,48]. The pyroptosis inhibitory effect of notopterol may have therapeutic implications for these diseases.

This study contained some limitations. First of all, we performed experiments in H9c2 cell lines, in which existed subtle differences compared to primary cells. Therefore, future investigation needs to be performed in primary cells. Moreover, there could be alternative pathways that participate in hyperuricemia-associated cardiac dysfunction besides pyroptosis and inflammation and other possible mechanisms require further investigation. Next, the serum uric acid concentrations in mice are much lower than that in humans, and it is difficult to induce mice with equivalent serum uric acid concentrations to hyperuricemia in humans. Finally, the mechanism on the effect of allopurinol and notopterol on exercise capacity still needs further investigation.

## 4. Materials and Method

### 4.1. Animals and Reagents

All animal experiments were approved by the Animal Care and Use Committee of Tongji Hospital, Tongji Medical College, Huazhong University of Science and Technology (Approval code: TJH-202205004). Eight-week-old male wild-type C57BL/6J mice (about 22 g) were purchased from the Shulaibao Biotech. After an adaptation period of one week, the mice were randomly divided into four groups with five in each group: Control, Hyperuricemia, Hyperuricemia + Allopurinol (10 mg/kg/day), Hyperuricemia + Notopterol (20 mg/kg/day). The mouse model of hyperuricemia was induced by gavage of a mixture of adenine (160 mg/kg) (HY-B0152, MedChemExpress, Princeton, NJ, USA) and potassium oxonate (2400 mg/kg) (HY-17511, MedChemExpress) dissolved in 0.5% carboxymethycellulose sodium (CMC-Na) every other day for six weeks. The control group was given the same amount of 5% CMC-Na by gavage. One week after hyperuricemia induction, blood samples were harvested from the caudal vein of mice and centrifuged to separate the serum to detect serum uric acid. Two weeks after hyperuricemia induction, the mice in the Hyperuricemia + Allopurinol group were given allopurinol (HY-B0219, MedChemExpress) at 10 mg/kg dissolved in 0.5% CMC-Na daily by gavage for another four weeks. Meanwhile, the mice in the Hyperuricemia + Notopterol group were administrated notopterol (SN8250, Lot.No.505D021, Sorlabio, Beijing, China) 20 mg/kg by gavage for four weeks. Hyperuricemia and control groups were administered with an equal amount of 0.5% CMC-Na in parallel. After six-weeks feeding, all animals were euthanized and the cardiac tissues were collected for histologic examination and protein expression analysis. Serum samples were collected for the measurement of serum uric acid. Serum uric acid levels were detected by Uric acid Test Kit (C012, Nanjing Jiancheng bioengineering institute, Nanjing, China).

### 4.2. Treadmill Fatigue Test

Treadmill Fatigue Tests were performed at the sixth week. The mice were acclimated to treadmill running (about 20 mins/day) over 3 days. The maximum treadmill (ZS-PT-III, Zhongshi technology, Shenzhen, China) incline was 20°. The initial speed of the treadmill fatigue test was 5 m/min for 4 min, then the speed was increased to 14 m/min for 2 min and with 2 m/min increments every minute until exhaustion, which was considered to be when the mouse remained in the fatigue zone for 10 continuous seconds despite mild electrical prodding (0.6 mA). The electrical prodding was turned off when the mouse showed exhaustion. The running time and running distance were both recorded.

### 4.3. Echocardiography

Echocardiography was performed at the second week and at the sixth week using a VINNO6 high resolution imaging system (VINNO Corporation, Suzhou, China). Mice were anesthetized with 1.5–2% isoflurane and the chests were shaved. Then, mice were placed in a supine position and ultrasound gel was applied to the chest. Afterward, mice were subjected to echocardiographic examination. M-mode tracings were recorded from the short-axis view at the papillary muscle level of the left ventricle. The parameters of cardiac function included left ventricular ejection fraction (LVEF), left ventricular fractional shortening (LVFS), left ventricular volumes at end diastole (LVEDV) and left ventricular volumes at end systole (LVESV). The cardiac parameters were acquired from at least three separate cardiac cycles. The group of mice was blinded to echocardiographic operators.

### 4.4. Immunohistochemistry

The heart tissue was removed and fixed with 10% formalin after the mice were sacrificed. Then the fixed myocardial tissue was embedded in paraffin and sectioned. Immunohistochemistry for protein expression was performed with antibodies against IL-18 (A20473, Abclonal, Wuhan, China), IL-1β (A19635, Abclonal, Wuhan, China), and NLRP3 (19771, Proteintech, Wuhan, China). Image capture was performed using an MShot microscope (Wuhan, China).

### 4.5. Cell Culture and Treatment

H9c2 cells were purchased from the American Type Culture Collection (ATCC, Manassas, VA, USA). Cell culture was performed in high glucose Dulbecco’s modified Eagle’s medium (DMEM, KeyGEN BioTECH, Nanjing, China) supplemented with 10% fetal bovine serum (FBS, Gibco, Waltham, MA, USA) and 1% penicillin/streptomycin (Sangon, Shanghai, China) at an atmosphere of 37 °C with 5% CO_2_. Cells were induced with uric acid (U2625, Sigma, St. Louis, MO, USA; Kawasaki, Japan) for 24 h to simulate high uric acid conditions. To illustrate the effects of notopterol, H9c2 cells were treated with notopterol (25 μM) incubated with uric acid (200 mg/L) for 24 h and then cells were harvested for western blotting.

### 4.6. Compounds

Urid Acid was purchased from Sigma-Aldrich (U2625, St. Louis, MO, USA), with purity (HPLC) ≥99%. The powder of uric acid was dissolved in 1 M NaOH solution at a concentration of 45 mg/mL. Notopterol was purchased from Sorlabio (SN8250, Beijing, China), with purity (HPLC) > 98% and dissolved in dimethyl sulfoxide (DMSO) at a concentration of 10 mM. Brilliant Blue G was purchased from Sorlabio (BBG, C8420, Beijing, China), with purity (HPLC) > 98% and was dissolved in DMSO at a concentration of 10 mM.

### 4.7. Cell Viability Assay

Cell activity was measured with a CCK8 assay kit. Briefly, H9c2 cells were counted using a cell counter (Nexcelom Biosciences, Lawrence, KS, USA) and were seeded in 96-well plates (5000 cells/well). After 12 h in culture, cells were treated with notopterol (10, 25, 50, and 100 μM) for 24 h. CCK8 solution was incubated with cells for 2 h at 37 °C protected from light. The absorbance was measured at a wavelength of 450 nm using a spectrophotometer.

### 4.8. Real-Time Fluorescence Quantification PCR

Total RNA was obtained from heart tissues and H9c2 cells. The RNA quality and concentration were detected spectrophotometrically (NanoDrop 2000 spectrophotometer, Thermo Scientific, Waltham, MA, USA). PrimeScript™ RT Master Mix (Takara, Kusatsu, Japan) was used to synthesize cDNA. Real-time fluorescence quantification PCR was performed to detect the levels of target genes with the SYBR™ Select Master Mix (Thermo Fisher). Quantification of IL-1β and IL-18 in heart tissue and H9c2 cells were normalized to Rn18s and GAPDH by the ΔΔCT method, respectively. The primers were rat GAPDH forward AGGTCGGTGTGAACGGATTTG, reverse TGTAGACCATGTAGTTGAGGTCA. Rat IL-1β forward CCTCACCCTGTTTGGGGTTT, reverse GTTAGCATGCCTGCCCTGAA. Rat IL-18 forward CGACCGAACAGCCAACGAATCC, reverse TGTCCTGGCACACGTTTCTGAAAG. Mouse P2X7R forward GGTGGGGTGACGAAGTTAGG, reverse ATACTCAGGACACAGCGTCT. Mouse Rn18s forward GGACACGGACAGGATTGACAGATTG, reverse TAACCAGACAAATCGCTCCACCAAC. Mouse IL-1β forward AATGAAGGAACGGAGGAGCC, reverse CTCCAGCCAAGCTTCCTTGT. Mouse IL-18 forward GACAGCCTGTGTTCGAGGAT, reverse TCCTTCACAGAAGGGTCACA. The expression level of target genes was presented as relative fold change with respect to the control group.

### 4.9. Western Blot

Heart tissues and the treated H9c2 cells were lysed using RIPA buffer (Sangon, Shanghai, China) with 1 mM protease inhibitor and 1 mM phosphatase inhibitor. Protein concentrations were measured by BCA protein assay (Sangon, Shanghai, China) and equal amounts of proteins (20–30 μg) were subjected to SDS-PAGE gels. A pre-stained protein ladder (26616, Thermo Scientific) was also applied as well. Proteins underwent electrophoresis at 60 V for 40 min and then this was turned to 120 V for 1 h. The separated proteins were transferred onto the PVDF membranes. After blocking with 5% skimmed milk for 90 min, membranes were incubated with primary antibody at 4 °C overnight, including P2X7R (A10511, Abclonal, Wuhan, China, 1:1000), NLRP3 (19771, Proteintech, Wuhan, China, 1:1000), Caspase-1 (A16792, Abclonal, Wuhan, China, 1:1000), Caspase 1 (22915, Proteintech, Wuhan, China, 1:1000), IL-1β (A19635, Abclonal, Wuhan, China, 1:1000), IL-18 (A20473, Abclonal, Wuhan, China, 1:1000), β-tubulin (AC021, Abclonal, Wuhan, China, 1:5000). At room temperature, the membranes were incubated with horseradish peroxidase-labelled secondary antibodies and ultrahigh sensitivity ECL kit (HY-K1005; MedChemExpress). The information of secondary antibodies are as follows: HRP Goat Anti Mouse IgG (HA1006, Promoter), HRP Goat Anti Rabbit IgG (HA1005, Promoter). Protein detection was performed on the ChemiDoc-It 510 Imager with VisionWorks software (Ultra-Violet Products Ltd., Cambridge, UK).

### 4.10. Immunofluorescence Staining

H9c2 cells were cultured for 12 h and were treated with stimulation for 24 h. Treated cells were fixed with 4% paraformaldehyde for 15 min at room temperature. Cells were washed twice with PBS and were blocked with 5% goat serum and 0.5% Triton. Subsequently, the cells were incubated with primary antibodies targeting P2X7R (A10511, Abclonal, Wuhan, China) overnight at 4 °C and further incubated with secondary antibody for 1 h. Finally, the glass slides were sealed with the DAPI-containing anti-fluorescence quencher. Slides were examined and photographed using a confocal microscope (Nikon confocal microscope, C2+, Nikon, Tokyo, Japan).

### 4.11. Transfection

For siRNA transfection, H9c2 cells were cultured in 6-well culture plate for 12 h. Then the cells were transfected with control siRNA or P2X7R siRNA (RiboBio, Guangzhou, China) using Lipofectamine 2000^®^ (11668019; Invitrogen, Waltham, MA, USA). Briefly, seeded H9c2 cells were cultured in mixed Opti-MEM^®^ I reduced serum medium (#31985070; Gibco; Thermo Fisher Scientific, Inc., Waltham, MA, USA) containing 50 nM siP2X7R and Lipofectamine 2000^®^ in line with the instructions. Twenty-four hours later, H9c2 cells were treated with uric acid for 24 h.

For overexpression, H9c2 cells were cultured in 6-well culture plate for 12 h and transfected with control plasmid or P2X7R overexpressing plasmid using Lipofectamine 3000^®^ (L3000015; Invitrogen) in Opti-MEM^®^ I reduced serum medium. H9c2 cells were stimulated with uric acid and notopterol for 24 h post-transfection and then cells were harvested for western blotting.

### 4.12. Statistical Analysis

GraphPad prism 8.0 and SPSS 28.0 were used for drawing and statistical analysis. Data were shown as mean ± S.E.M in this experiment. The normal distribution of the data was checked using the Shapiro-Wilk test in our study. Statistical analysis was performed using unpaired Student’s *t*-test (equal vari-ance) or one-way analysis of variance (ANOVA) for normal distributed data. The Mann-Whitney U test or the Kruskal-Wallis test was used to compare nonnormally distributed data. *p* < 0.05 was defined as significant.

## 5. Conclusions

**Figure 8:**
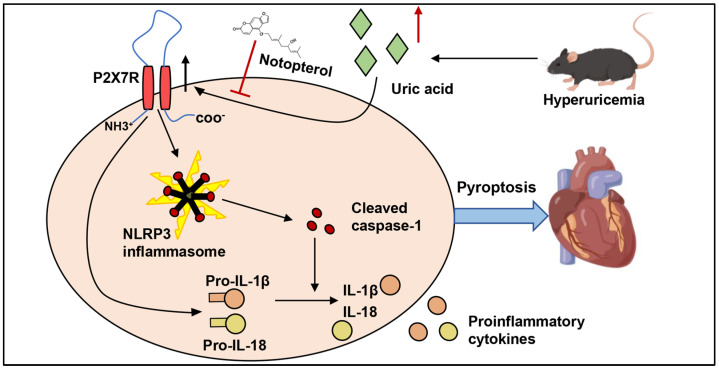
Summary of the findings of this study and schematic of mechanisms of Notopterol alleviate pyroptosis and inflammation in cardiac tissue of hyperuricemic mice. P2X7R played an important role in NLRP3 inflammasome activation and IL-18 and IL-18 production induced by uric acid. Notopterol inhibited P2X7R expression and suppressed NLRP3 inflammasome activation, inhibting the cleavage of caspase1, attenuated pyroptosis and inflammation in cardiac tissue of hyperuricemic mice. Black arrows indicate activation or promotion, red arrows represent upregulation. red T-shaped lines indicate inhibition.

Notopterol improved exercise capacity, attenuated cardiac dysfunction, suppressed the cardiac inflammatory response and attenuated pyroptosis in the cardiac tissue of hyperuricemic mice. P2X7R was an upstream regulator of pyroptosis and inflammatory cytokine production in H9c2 cells under uric acid stimulation. Notopterol inhibited pyroptosis via suppressing the P2X7R/NLRP3 signaling pathway (Figure 8). Notopterol might represent a potential therapeutic strategy against pyroptosis and improve cardiac function in hyperuricemic mice.

## Data Availability

Data is contained within the article.

## Supplementary Materials

The following supporting information can be downloaded at: https://www.mdpi.com/article/10.3390/ph16030361/s1. Supplementary Figure S1. The chemical structure of Notopterol. Supplementary Figure S2. Experimental design scheme and cardiac function measured at the second week after hyperuricemia induction. (A) The flowchart showing the experimental process for modeling mice. (B) Left ventricular ejection fraction (LVEF), (C) Left ventricular fractional shortening (LVFS), (D) Left ventricular volumes at end diastole (LVEDV) and (E) systole (LVESV) were measured at the sixth week after hyperuricemia induction (n = 5 mice/group). Data represent means ± S.E.M. The symbol “*” represents *p* < 0.05 vs. control. The “ns” stands for “no significance”.
