# Supplementary material for: Notopterol Ameliorates Hyperuricemia-Induced Cardiac Dysfunction in Mice
Source: Pharmaceuticals (Basel). 2023 Feb 27;16(3):361. doi: 10.3390/ph16030361 (PMC10052463; doi:10.3390/ph16030361)
Supplement: Supplementary file 1 [file pharmaceuticals-16-00361-s001.zip › Supplementary Figures.pdf]

Supplementary Figures

1. Supplementary Figure

Supplementary Figure S1.

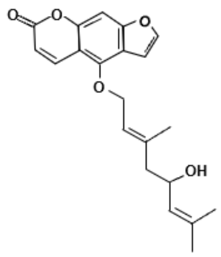

Figure S1. The chemical structure of Notopterol.

Supplementary Figure S2.

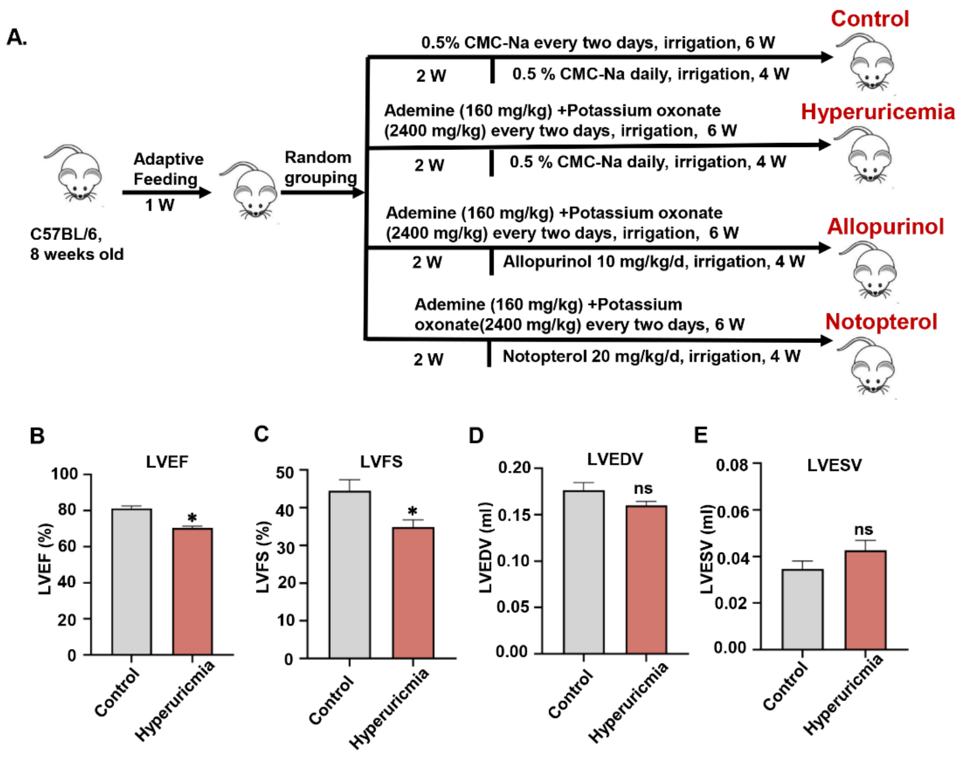

Figure S2. Experimental design scheme and cardiac function measured at the second week after hyperuricemia induction. (A) The flowchart showing the experimental process for modeling mice. (B) Left ventricular ejection fraction (LVEF), (C) Left ventricular fractional shortening (LVFS), (D) Left ventricular volumes at end diastole (LVEDV) and (E) systole (LVESV) were measured at the sixth week after hyperuricemia induction (n=5 mice/group). Data represent means ± S.E.M. The symbol "*" represents $P < 0.05$ vs. control. The "ns" stands for "no significance".
