# Supplementary material for: Notopterol Ameliorates Hyperuricemia-Induced Cardiac Dysfunction in Mice
Source: Pharmaceuticals (Basel). 2023 Feb 27;16(3):361. doi: 10.3390/ph16030361 (PMC10052463; doi:10.3390/ph16030361)
Supplement: Supplementary file 1 [file pharmaceuticals-16-00361-s001.zip › Supplementary Material.pdf]

Data of Figure 2A (n=5)

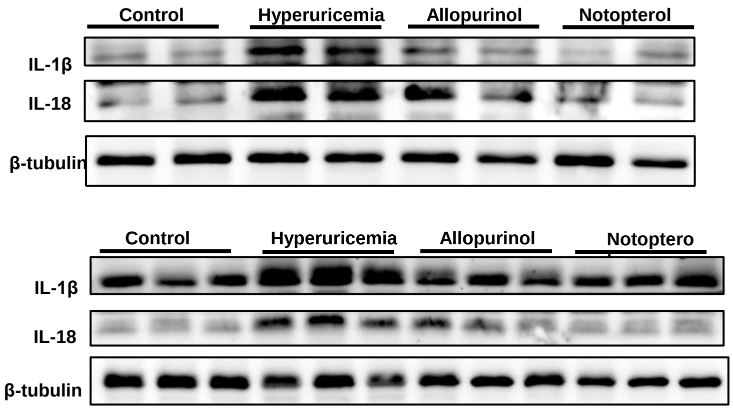

Data of Figure 3A (n=5)

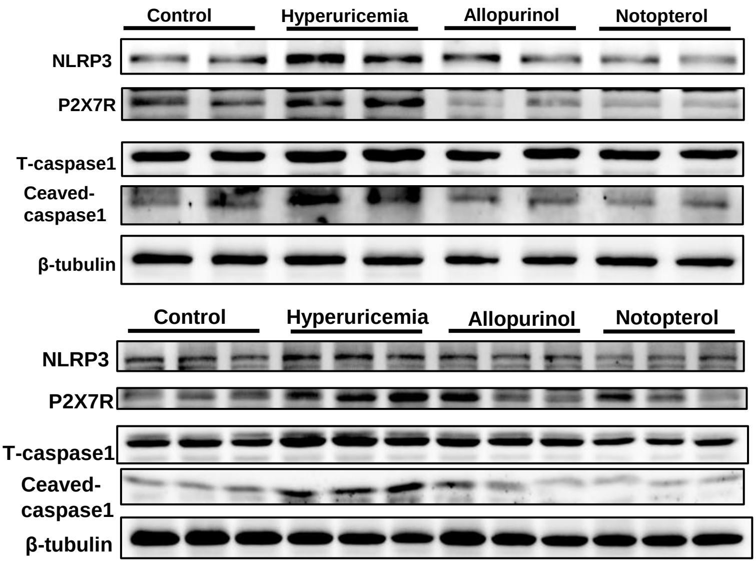

Data of Figure 4A (n=3)

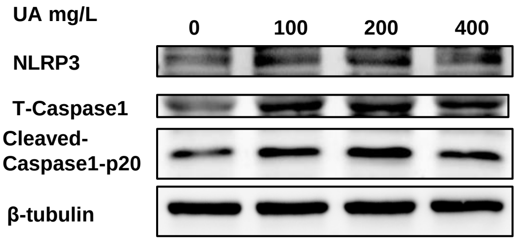

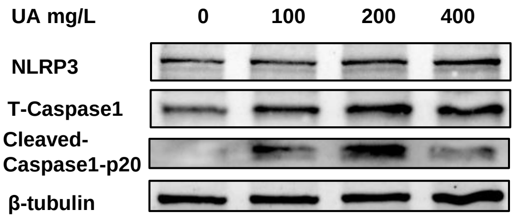

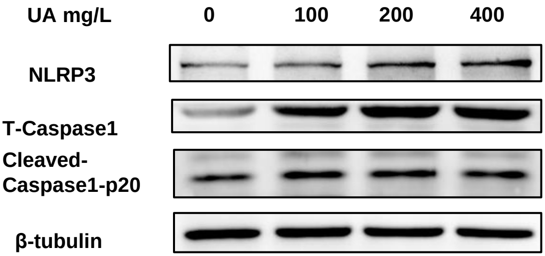

Data of Figure 4G (n=3)

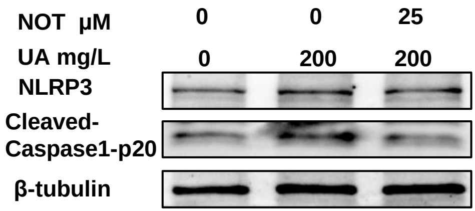

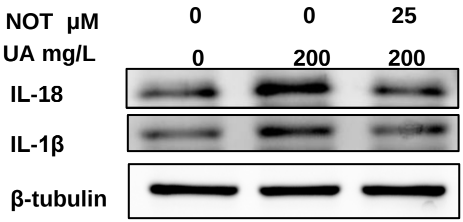

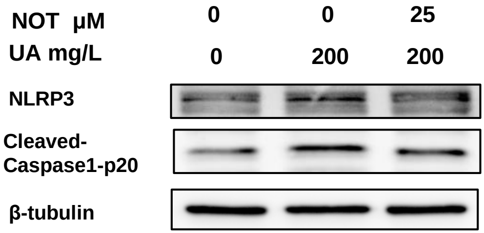

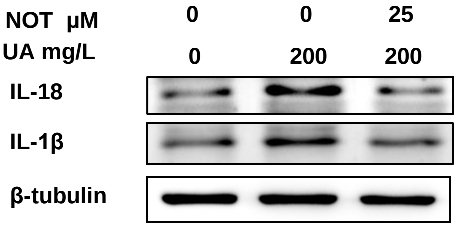

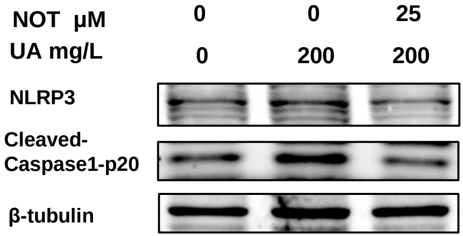

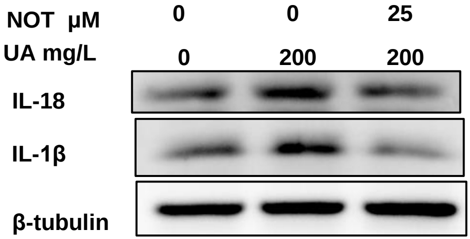

Data of Figure 5A (n=3)

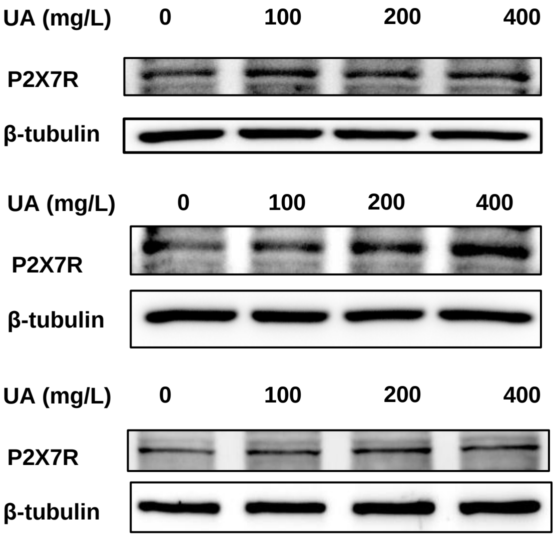

Data of Figure 5C (n=3)

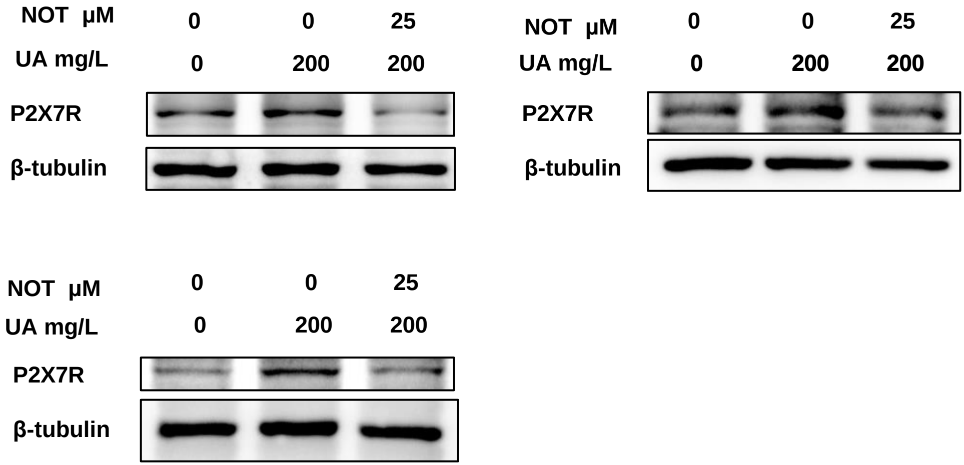

Data of Figure 6A (n=3)

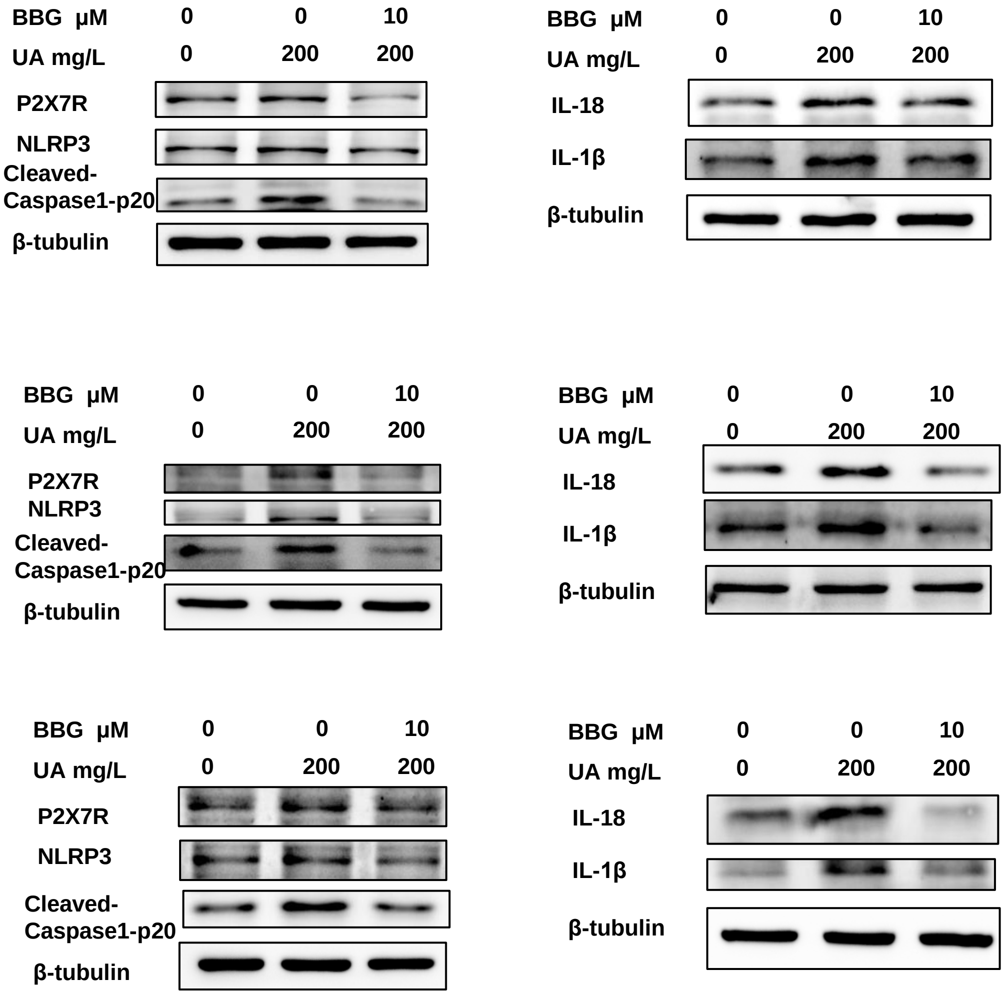

Data of Figure 6D (n=3)

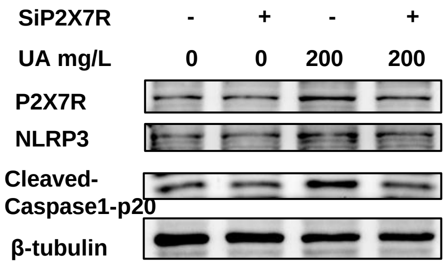

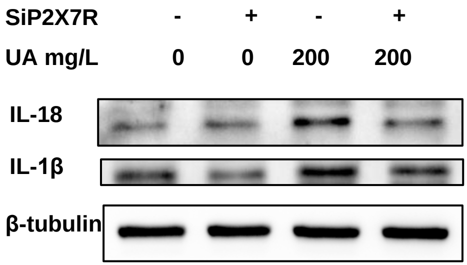

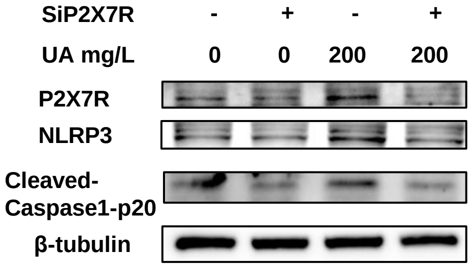

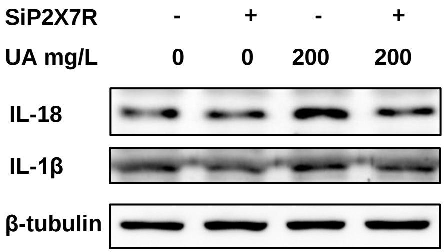

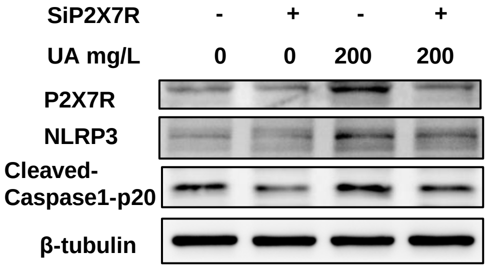

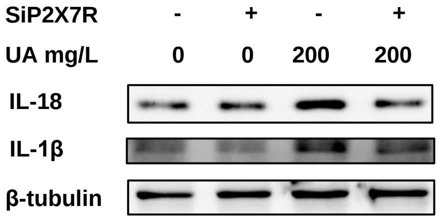

Data of Figure 7A (n=3)

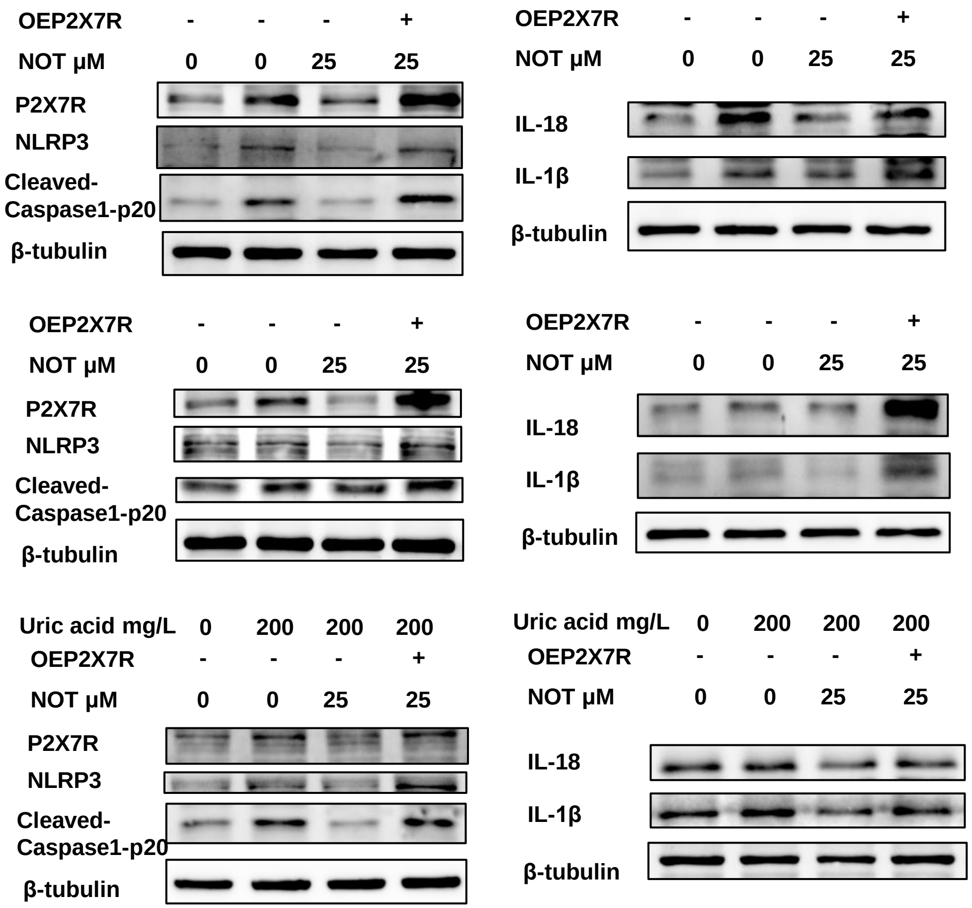

Internal positive controls of antibodies

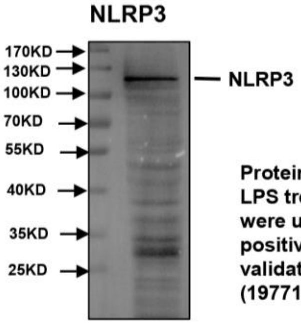

Proteins extracted from LPS treated THP-1 cells were used as internal positive controls to validate NLRP3 abtibody. (19771, Proteintech)

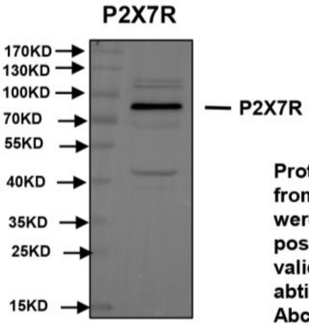

Proteins extracted from lung tissue of rat were used as internal positive controls to validate P2X7R abtibody. (A10511, Abclonal)

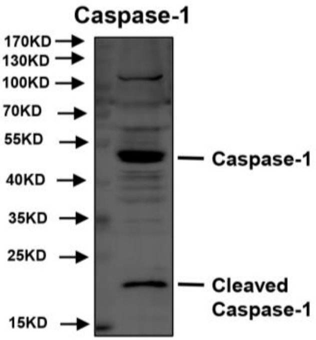

Proteins extracted from LPS treated THP-1 cells were used as internal positive controls to validate Caspase-1 abtibody. (A16792, Abclonal)

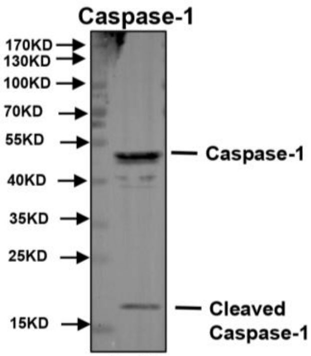

Proteins extracted from LPS treated THP-1 cells were used as internal positive controls to validate Caspase-1 abtibody. (22915, Proteintech)

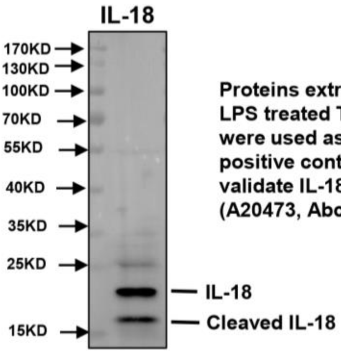

Proteins extracted from LPS treated THP-1 cells were used as internal positive controls to validate IL-18 antibody. (A20473, Abclonal)

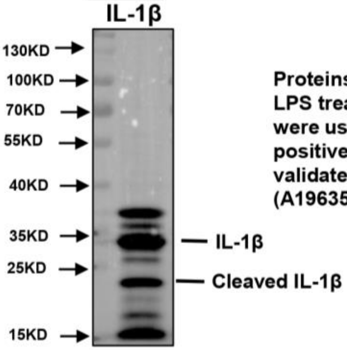

Proteins extracted from LPS treated THP-1 cells were used as internal positive controls to validate IL-1β antibody. (A19635, Abclonal)
